# Local Exposure to Recombinant Human Bone Morphogenetic Protein-2 in Spinal Fusion: Limitations of Total Mass and a Proposed Anatomic Treatment Unit Dose Concentration

**DOI:** 10.7759/cureus.114637

**Published:** 2026-08-17

**Authors:** John P Von Benecke

**Affiliations:** 1 Research, Locate Bio Ltd, Nottingham, GBR

**Keywords:** anatomic treatment unit, atu dose concentration, local safety, rhbmp-2, spinal fusion, total mass

## Abstract

Recombinant human bone morphogenetic protein-2 (rhBMP-2) is effective for promoting spinal fusion, but local safety is commonly interpreted from total mass in milligrams. This structured narrative review examines the limitations of total mass and a proposed graft-normalized concentration measure that relates rhBMP-2 mass to the complete graft construct within one anatomic treatment unit (ATU). PubMed was searched on June 27, 2026, for records published from June 1, 2016 through June 26, 2026, using ((((rhBMP-2) OR (recombinant human bone morphogenetic protein-2)) AND (spine)) NOT (Maxillofacial)) NOT (Dental). The search returned 253 records. The evidence base was supplemented by backward citation searching and targeted foundational, mechanistic, anatomical, graft-volume, and regulatory sources, together with one post-search source. In interbody fusion, one ATU is one treated motion segment after cage placement. The product loading concentration, such as 1.5 mg/mL for Infuse Bone Graft (Medtronic; Memphis, Tennessee, USA), describes rhBMP-2 on its carrier before implantation. ATU dose concentration is the rhBMP-2 mass per ATU divided by the total graft volume implanted in that ATU. It is a construct-average descriptor, not a measured tissue concentration; realized exposure also depends on retention, release, mixing, spatial distribution, placement, containment, and local tissue anatomy. Across the reviewed evidence, total mass alone did not provide a sufficient general description of local biological and spatial outcomes when graft configuration, delivery, or anatomy changed. Higher nominal-dose comparisons usually changed other exposure variables, whereas equal-mass experiments produced different biological or spatial outcomes when concentration or delivery changed. These findings support reporting the completed graft construct and local anatomical context alongside mass, but they do not establish total graft volume as the biologically correct denominator or validate ATU dose concentration as an independent predictor of clinical adverse events. ATU dose concentration is therefore presented as a proposed complementary exposure descriptor requiring direct patient-level validation.

## Introduction and background

Recombinant human bone morphogenetic protein-2 (rhBMP-2) is a potent osteoinductive protein used to augment spinal fusion [1-3]. Contemporary reviews support its efficacy in selected lumbar, deformity, and revision settings while emphasizing procedure-specific risks, cost, and the importance of dose and delivery [1,2]. In the United States, Infuse Bone Graft (Medtronic; Memphis, Tennessee, USA) was originally approved in 2002 as a system comprising rhBMP-2, an absorbable collagen sponge, and a specified anterior lumbar interbody device [3]. In February 2026, the indication was expanded to one- and two-level transforaminal lumbar interbody fusion from L2 to S1 when used with an eligible interbody device and supplemental fixation [4].

Much of the clinical safety literature reports rhBMP-2 exposure as total mass or mass per level [5-10]. Reports of cervical swelling and airway compromise, vertebral osteolysis, radiculitis, heterotopic ossification, seroma, and other local complications led to intensified concern about rhBMP-2 exposure [5-7] and contributed to subsequent dose reduction in clinical practice [8-10]. A longitudinal cohort reported that rhBMP-2 dose reduction was associated with fewer complications [8]. A meta-analysis of posterior lumbar interbody fusion reported high fusion rates across a broad dose range but found no consistent relationship between dose and complications [9]. Separately, institutional data showed that rhBMP-2 dosing for interbody fusion declined over time [10]. Using less rhBMP-2 can be a sensible clinical intervention, but dose reduction changes the treatment package and does not establish total mass as the unique local safety variable.

The contrast is clearest in interbody fusion. Product loading concentration describes the rhBMP-2 concentration on its carrier before other graft is added [3,4]. At a fixed rhBMP-2 mass, additional graft containing no rhBMP-2 lowers the nominal whole-construct average to the extent that it increases the final implanted graft bulk volume [4,11]. The term ATU dose concentration is used here for rhBMP-2 mass at one treated level divided by the total graft volume implanted at that level. Patient anatomy and cage geometry constrain the graft-bearing volume [4,11,12], while placement and graft distribution define local tissue relationships [4,13]. The absorbable collagen sponge (ACS) is soft and pliable and may deform during preparation, handling, and implantation; accordingly, nominal prepared volume may not equal its implanted bulk volume contribution [3,14].

This concern is not new. Boden et al. stated that, rather than overall dose alone, the parameter appearing most predictive of osteoinductive efficacy was protein concentration expressed as milligrams per unit volume of the total implant. They described a threshold concentration below which bone induction was absent or variable and above which consistent bone induction could be observed, with that threshold varying by osteoinductive factor, carrier matrix, anatomical location, species, and systemic healing conditions. Above the threshold, implant volume was described as determining the amount of bone formed and carrier shape as determining its geometry [15]. The original Food and Drug Administration (FDA) Summary of Safety and Effectiveness Data (SSED) similarly described bone formation in relation to rhBMP-2 concentration and residence time at the implant site [3].

The present review examines whether total rhBMP-2 mass alone adequately describes nominal local exposure when graft volume, delivery, and anatomy vary, and evaluates whether ATU dose concentration may provide a complementary exposure descriptor. The review specifically considers evidence in which mass changes together with other exposure variables, evidence in which mass is held constant while concentration or delivery changes, and evidence relevant to anatomical or cross-species transportability. ATU dose concentration is treated throughout as a proposed construct-level measure, not as a measured tissue concentration or a validated predictor of clinical adverse events.

## Review

Search strategy and analytic approach

A structured narrative search of PubMed was performed on June 27, 2026, using the query ((((rhBMP-2) OR (recombinant human bone morphogenetic protein-2)) AND (spine)) NOT (Maxillofacial)) NOT (Dental). Results were limited to publication dates from June 1, 2016, through June 26, 2026. The search returned 253 records. Titles and abstracts were screened for studies relevant to the review question, including clinical or preclinical reports describing rhBMP-2 mass, loading concentration, carrier volume, implanted graft volume, placement, containment, release, local adverse effects, or exposure-response relationships. Each record was assigned one of four dispositions according to its relationship to the review question and incremental value. Records were classified as excluded at title/abstract when they did not address the predefined exposure domains; eligible but redundant when they met broad relevance criteria but duplicated an exposure contrast already represented by a more direct, better-reported, or more clinically relevant source; relevant but not discussed when they informed the exposure framework but did not materially alter the narrative synthesis; or included in the narrative when they materially informed a principal exposure contrast or limitation. Of the 253 records, 119 were excluded at title/abstract screening; 85 were eligible but redundant; 25 were relevant to the exposure framework but not individually discussed; and 24 materially informed the narrative synthesis. The evidence base was supplemented by backward citation searching and by targeted retrieval of pharmacokinetic evidence [16], relevant regulatory documents [17,18], nonspinal carrier and delivery studies [19-23], foundational studies published before the search interval, anatomical and graft-volume studies, and one directly relevant post-search publication [24]. These additional sources were included when they addressed an evidentiary gap not adequately covered by the contemporary PubMed search. Sources materially informing the narrative synthesis are summarized separately in Appendix A, together with their study design, evidentiary role, relevance to the review question, principal limitations, and design-appropriate internal validity considerations. The sole author performed the literature search, title and abstract screening, record classification, full-text assessment of sources materially informing the narrative, source selection, data extraction, qualitative appraisal, and narrative synthesis. Clinical directness reflected proximity to human spinal application and was considered separately from methodological quality or evidence certainty. 

The review was designed to test a mass-only interpretation rather than estimate a pooled treatment effect. The evidence discussed spans clinical, preclinical, regulatory, anatomical, and mechanistic sources. For the purposes of interpretation, the narrative distinguishes between direct evidence, in which mass was held constant while concentration, carrier, release, or spatial presentation changed; coupled evidence, in which mass changed together with product loading concentration, carrier volume, graft composition, anatomy, practice, or more than one of these variables; and contextual evidence, comprising cross-procedure, cross-anatomical or cross-species comparisons relevant to the transportability of nominal mass. Throughout the review, these different evidence types are interpreted according to their design, directness to the review question, and principal limitations. Appendix A summarizes the principal sources discussed in the narrative synthesis, their evidentiary role, and design-specific internal validity considerations where these materially affect interpretation. Because the review draws on heterogeneous evidence and was not designed to estimate a pooled treatment effect, no single formal risk-of-bias or certainty framework was applied across the entire evidence base. Because this was a structured narrative review rather than a systematic review of intervention effects, PRISMA was not applied; the record-level audit was provided to improve transparency. Numerical findings retain each source’s original definitions and denominators.

The key test is straightforward. A higher-dose group cannot isolate the effect of milligrams if it also differs in product loading concentration, loaded-carrier volume, additional graft volume, delivery, or anatomy. Conversely, different outcomes at the same mass show directly that mass alone is insufficient. The review then examines whether ATU dose concentration may provide a useful complementary description of nominal local exposure, while recognizing that its biological and clinical validity has not been established. Total mass may remain useful within a tightly standardized product, anatomy, and surgical technique.

Interbody ATU and dilution principle

Throughout this review, total dose means nominal rhBMP-2 mass unless otherwise specified. Patient total mass and local mass should not be used interchangeably. For interbody fusion, one ATU is one treated motion segment after cage placement, including the cage graft chamber and any prespecified extracage graft bed. In multilevel surgery, each treated level is a separate ATU. The unit is anatomical because its graft-bearing volume and tissue relationships are determined by the patient, spinal level, device, and placement [4,12,13]. Where graft is distributed between distinct cage and extracage compartments, those compartments should also be reported separately rather than inferred from the whole-ATU value alone.

The product loading concentration describes rhBMP-2 in the loaded carrier before other graft is added. For Infuse, this is 1.5 mg/mL [3,4]. The completed construct may also contain autograft, allograft, ceramic, demineralized bone matrix, or other graft without rhBMP-2 [4,11].

ATU dose concentration is rhBMP-2 mass in one ATU divided by the total graft volume implanted in that ATU.

This measure captures construct-level normalization when the rhBMP-2-loaded carrier and additional graft are considered as components of the completed graft construct. If 1 mL of carrier loaded at 1.5 mg/mL is combined with 1 mL of graft containing no rhBMP-2 and the final implanted bulk volume is 2 mL, the complete construct contains 1.5 mg and its nominal ATU dose concentration is 0.75 mg/mL. At fixed rhBMP-2 mass, additional graft lowers the nominal construct-average concentration only to the extent that it increases the measured or estimated final implanted graft bulk volume. Where components are physically admixed, this may also represent greater physical dilution; spatially separated components may retain different local concentrations within the ATU.

Here, "dilution" is a construct-level description and should not be interpreted as requiring homogeneous physical mixing. The carrier and additional graft may be admixed, layered, fragmented, or spatially separated. ATU dose concentration is therefore an average across the completed implanted graft construct, not the concentration at every point within it. Where components occupy distinct compartments, the whole-ATU value should be interpreted together with the location and volume of the rhBMP-2-containing component rather than as evidence that all tissues within the ATU experience the same concentration.

Realized tissue exposure may differ from this average because rhBMP-2 retention and release depend on the carrier and delivery system [3,16,19-25]. Exposure is also influenced by mixing and spatial distribution, graft placement, and containment [4,13,19-26]. Anatomical dimensions, proximity to susceptible tissues, and procedure-specific graft-bed context further influence the clinical implications of local exposure [12,27-32]. Preclinical studies in nonhuman primate and ovine models document local resorptive and ectopic-bone responses under different implantation conditions [33,34]. Comparative anatomy and methodological reviews document cross-species differences in graft-space geometry and implant fit [35-37].

**Figure 1:**
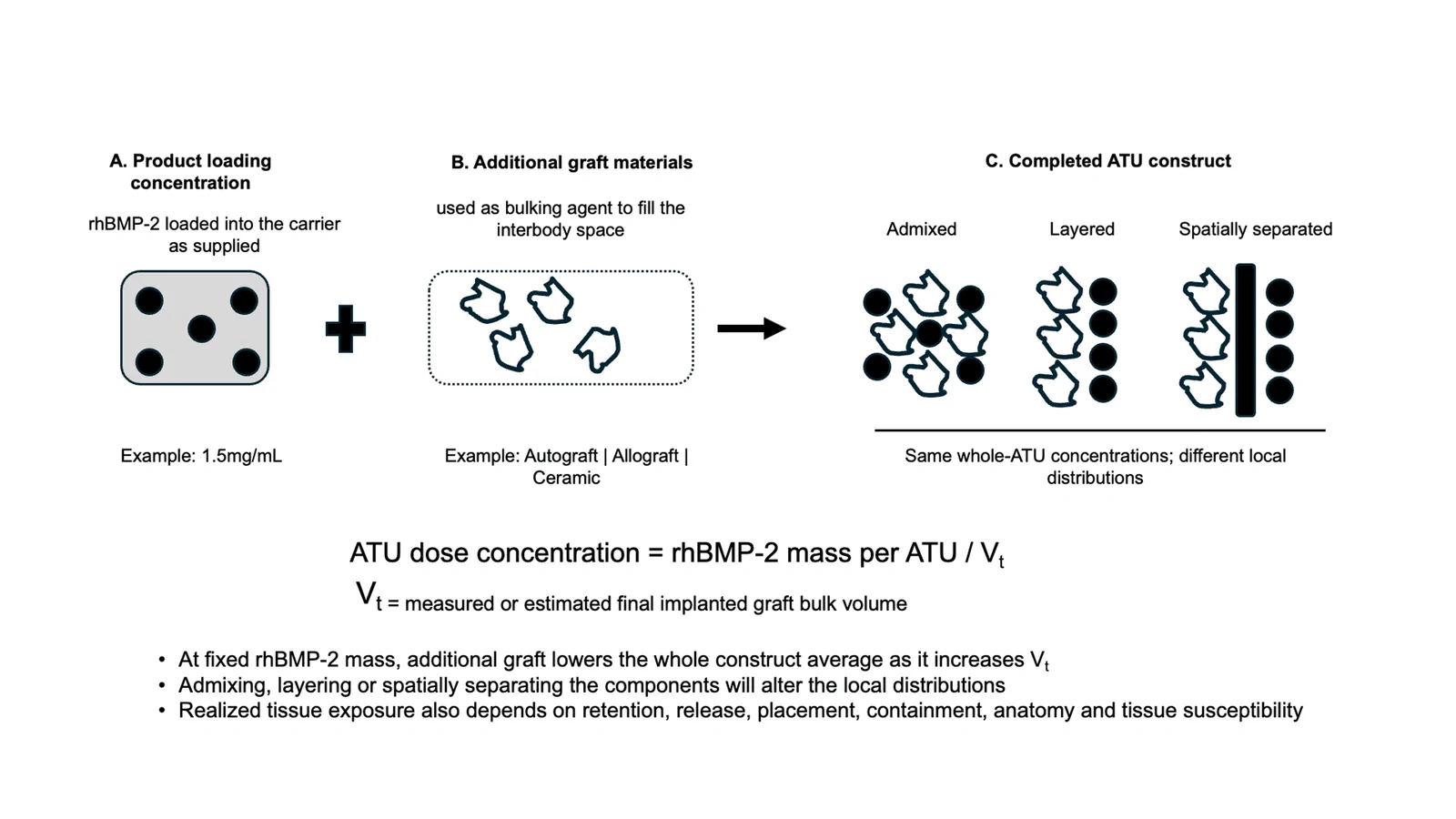
Product loading and nominal whole-construct concentration. Conceptual representation created by the author using Microsoft PowerPoint (Version 16.111.1) of product loading concentration and nominal whole-construct normalization. (A) Product loading concentration: rhBMP-2 loaded into the carrier as supplied. (B) Additional graft materials: graft materials without rhBMP-2 used as a bulking agent to fill the interbody space. (C) Completed ATU construct: the completed graft construct, illustrating admixed, layered, and spatially separated configurations. Product loading concentration describes rhBMP-2 in the carrier before other graft is added. At fixed rhBMP-2 mass, additional graft containing no rhBMP-2 lowers the nominal whole-construct average only to the extent that it increases the measured or estimated final implanted graft bulk volume. Component volumes may not be simply additive after packing, compression, or interpenetration. Where components are admixed, the reduction may also represent physical dilution; where they are layered or spatially separated, the concentration within the rhBMP-2-containing compartment may be unchanged despite a lower whole-ATU average. The ACS is a soft, pliable carrier that may be compressed during implantation, so nominal kit volume may not equal its in situ contribution. ATU dose concentration is not a measured tissue concentration; component configuration and location should be reported alongside the whole-ATU value. Realized exposure is further shaped by retention and release, spatial distribution, placement and containment, and local anatomy and tissue susceptibility. Conceptual synthesis is based on product and regulatory data, graft-volume and graft-distribution studies, and carrier-delivery evidence [3,4,11-14,16,19-26]. ACS: absorbable collagen sponge; ATU: anatomic treatment unit; DBM: demineralized bone matrix; rhBMP-2: recombinant human bone morphogenetic protein-2

In interbody fusion, the selected graft-bearing space is constrained by patient anatomy and cage geometry and is typically grafted to promote graft-host contact [11-13]. Because ACS is soft and pliable and may deform during preparation, handling, and implantation, nominal prepared volume may not equal its implanted bulk-volume contribution [3,14]. ATU dose concentration should therefore be calculated from the graft volumes actually implanted, with each component reported separately. Where the rhBMP-2-loaded carrier and additional graft occupy distinct compartments, their locations and component volumes should also be recorded so that the whole-ATU average is not mistaken for a homogeneous local concentration. Material prepared but not implanted should not contribute to the denominator. This is represented in Figure 1 below.

Open posterolateral fusion is different. It has no cage-defined graft chamber, and additional carrier and graft can be distributed across a broader decorticated area [26,32]. For these procedures, treated area or an explicitly defined dispersal volume may be more informative than a single volume denominator.

The various terms used, their plain-language definitions, and importance are set out in Table 1 below.

**Table 1:** Core exposure terms and nominal whole-construct concentration for an interbody anatomic treatment unit. Source support for product presentation, graft-volume context, and exposure modifiers [3,4,11-14,16,19-25]. Author-defined terms and the ATU dose concentration formula are proposed in this review. rhBMP-2: recombinant human bone morphogenetic protein-2; ATU: anatomic treatment unit; DBM: demineralized bone matrix

Term	Plain-language definition	Why it matters
Patient total mass	Sum of rhBMP-2 mass across all treated levels.	Needed for cumulative and systemic exposure; it does not describe local intensity.
Mass per ATU	rhBMP-2 mass assigned to one treated level.	Defines the local reservoir and should be reported per level.
Product loading concentration	rhBMP-2 concentration on the loaded carrier before other graft is added; 1.5 mg/mL for Infuse.	Describes the pre-implant product presentation, not the completed graft construct.
Additional graft	Autograft, allograft, ceramic, DBM, or other graft without rhBMP-2.	At fixed rhBMP-2 mass, lowers the nominal whole-construct average only to the extent that it increases final implanted graft bulk volume; local concentration also depends on configuration and spatial relationship to the rhBMP-2-loaded carrier.
Graft-bearing ATU	One treated motion segment after cage placement, including the intended cage and extracage graft bed.	Provides the local anatomical context and varies with patient anatomy and device choice.
Total implanted graft volume	Final implanted bulk volume occupied by the completed graft construct within the ATU, determined using a prespecified measurement convention. Nominal and implanted component volumes should be reported separately and should not be assumed to be additive when compression or interpenetration occurs.	Provides the denominator for whole-ATU nominal concentration; packing, compression, interpenetration, configuration, and measurement uncertainty should be reported.
ATU dose concentration	rhBMP-2 mass per ATU divided by total implanted graft volume.	A whole-construct nominal average; not a homogeneous or measured tissue concentration.
Realized local exposure	The exposure actually experienced by tissue within and around the graft.	Modified by retention, release, mixing and heterogeneity, placement, containment, and local anatomy.
Graft configuration	Spatial relationship of the rhBMP-2-loaded carrier to other graft components, for example admixed, fragmented, layered, cage-contained, or extracage.	The same whole-ATU average can represent different local concentration distributions when graft components are arranged differently.

The key test: does the same mass behave the same way?

A mass-only interpretation assumes that the same amount of rhBMP-2 has the same local meaning. If local exposure depends on more than mass alone, the same mass may behave differently when it is concentrated in less graft, more diluted by additional graft, retained or released differently, placed differently, or used in a smaller or more vulnerable anatomy.

This creates a simple evidentiary test. An association between higher nominal dose and an adverse outcome is not specific to milligrams when groups also differ in loading concentration, carrier volume, additional-graft volume, delivery, or anatomy. In contrast, different outcomes at the same mass show directly that total mass is insufficient. Such findings do not, by themselves, identify the correct alternative exposure metric or establish that ATU dose concentration predicts clinical adverse events.

What the evidence shows

Higher Nominal Dose Often Changes More Than Mass

Several frequently cited studies report more local adverse biology or more complications at higher nominal dose. In the ovine interbody study by Bae et al., the higher group received both greater mass and a higher loading concentration within a fixed device, and transient osteoclastic remodeling was greater [38]. In the ovine anterior lumbar interbody model reported by Pan et al., the groups also differed in both mass and loading concentration; cyst-like osteolytic voids occurred in both groups and were larger in the higher-exposure group [39]. These are genuine exposure-response findings, but they cannot distinguish the effect of mass from loading concentration or the combination of both. In these examples, no additional graft material was used, so there was no graft-dilution step and the reported loading concentration was the relevant nominal construct concentration [38,39].

The same problem applies clinically. De Stefano et al. reported an association between nominal dose per level and complications across 1,209 spinal arthrodeses [8]. With the commercial collagen-sponge system, using more product at the fixed 1.5 mg/mL product loading concentration changes both mass and nominal loaded-sponge volume [3,4]. In an interbody construct that also contains autograft, allograft, ceramic, demineralized bone matrix, or other graft, the resulting ATU dose concentration additionally depends on the volume of those components. Because patient-level graft volumes and configuration were not reported, the findings are consistent with fewer complications following adoption of a lower-dose treatment package but do not establish that total milligrams alone independently accounted for the local-safety signal [8].

The exploratory meta-analysis by Hofstetter et al. reported dose-complication relationships in some cervical and interbody strata but not consistently across procedures [26]. This heterogeneity suggests that a milligram placed in a smaller cervical graft space may not have the same local exposure implications as a milligram placed in a larger lumbar interbody construct or distributed across a posterolateral graft bed [12,26-32].

The transforaminal lumbar interbody fusion (TLIF)/posterior lumbar interbody fusion (PLIF) literature also varied substantially in the spatial presentation of rhBMP-2 [26]. Across the studies reviewed by Hofstetter et al. [26], the loaded carrier was placed anteriorly, within the cage, in both locations, or, in two studies, morselized with bone graft. A whole-ATU average can therefore conceal markedly different subcompartment exposures: a discrete loaded carrier can create an rhBMP-2-rich region adjacent to graft containing no rhBMP-2, whereas fragmentation can distribute rhBMP-2-bearing carrier over a larger surface area and into more locations. In a descriptive study-level comparison, Hofstetter et al. reported a higher frequency of symptomatic ectopic bone in the two studies in which BMP was mixed or morselized with bone graft than in studies placing it anteriorly or within the spacer [26]. Compartmentalized constructs were also associated with radiculitis, inflammatory cysts or seromas, osteolysis, and radiographic heterotopic bone [26]. Because rhBMP-2 mass, graft volume, placement, imaging surveillance, and adverse-event definitions differed between studies, these observations do not establish whether mixing or spatial concentration heterogeneity independently increased risk. They support reporting the completed graft configuration, including whole-ATU dose concentration, carrier configuration, cage or extracage placement, and proximity to vulnerable tissues, rather than interpreting total mass in isolation.

The 2026 Medtronic Infuse transforaminal lumbar interbody fusion program is another coupled comparison: rhBMP-2 mass and nominal loaded-sponge volume doubled together at fixed product loading concentration, while all subjects also received local autograft and optional allograft [4]. Because patient-level final implanted graft bulk volume and compartmental allocation were not reported, the magnitude of any whole-construct concentration difference cannot be reconstructed. The program therefore compared complete graft configurations rather than milligrams in isolation and is considered in detail in the regulatory case study below.

The Same Mass Can Produce Different Outcomes

If mass alone determined local biology, equal masses should behave similarly. Barnes et al. used the same 3 mg per side in a nonhuman primate posterolateral fusion model but changed final implant concentration and carrier configuration [40]. When the protein was distributed through a bulking matrix, the final concentration was 0.6 mg/mL and fusion failed. When the same 3 mg remained at 1.5 mg/mL on an absorbable collagen sponge wrapped around the same matrix, solid bilateral fusion occurred. The endpoint was efficacy, but the result shows plainly that the same mass did not have the same biological effect.

Contemporary spinal models extend this same-mass test. Hu et al. compared 100 µg rhBMP-2 delivered by a controlled-release polyelectrolyte complex or ACS in a rabbit interbody fusion chamber; the controlled-release construct fused whereas the equal-mass ACS construct did not, with differing osteogenic, inflammatory, and osteoclastic molecular profiles [41]. Chan et al. used 0.53 mg rhBMP-2 per side with the same ACS loading preparation in a split-side posterolateral fusion model; open versus percutaneous delivery produced different fusion and ectopic-bone outcomes despite equal mass and loading preparation [42]. These studies isolate release or delivery more directly than dose-response comparisons, although their endpoints remain preclinical.

Other contemporary spinal studies similarly show that equal nominal mass need not produce the same biological result. Cottrill et al. compared collagen sponge and BioMim-PDA at 0.2, 2.0, and 20 µg per side, demonstrating carrier-dependent differences in fusion and bone microarchitecture at matched doses [43]. Kim et al. compared 5 µg free rhBMP-2 with 5 µg delivered in calcium-binding PLGA/BMP microparticles and reported less ectopic bone with the bone-retaining delivery system while supporting fusion [44]. Refaat et al. found that 2 µg rhBMP-2 plus BMP-binding COMP outperformed 2 µg rhBMP-2 alone and approximated the 10 µg comparator; because COMP is biologically active, this is best interpreted as direct/coupled evidence that biological presentation can modify the mass-effect relationship [45].

Targeted nonspinal carrier and delivery studies provide complementary biological evidence. Each included a matched nominal-rhBMP-2-mass comparison in which carrier, retention, or release differed; some also included separate dose-response arms [19-23]. Collectively, they show that carrier, retention, and release can alter the amount or spatial distribution of regenerated or ectopic bone at the same nominal rhBMP-2 mass. These studies do not estimate clinical spinal complication rates, but they reinforce the broader conclusion that retention, release, and spatial presentation can modify local outcomes independently of a mass-only description.

Taken together, the evidence is asymmetric in an important way. Across higher nominal-dose comparisons, rhBMP-2 mass commonly changed together with one or more of product loading concentration, loaded-carrier volume, additional-graft volume or configuration, delivery, anatomy, and practice. Depending on the relative changes in mass and final implanted graft bulk volume, the resulting ATU dose concentration could increase, decrease, or remain unchanged. In the opposite direction, equal-mass studies produced different biological and spatial outcomes. These findings show that total mass alone is not a sufficient general description of local exposure, but they do not identify a single alternative metric that fully captures realized tissue exposure. They instead support reporting the completed graft construct and local anatomical context alongside mass. This asymmetric pattern is summarized in Table 2 below.

**Table 2:** Why total mass alone is insufficient as a general local-exposure descriptor. Summary of the pattern of results indicating that total mass alone is insufficient as a general descriptor of local exposure. Higher-dose comparisons usually changed several features together, whereas equal-mass studies showed that concentration and delivery can change biological outcomes [4,8,12,19-23,26-45]. Some studies also included dose-response arms. Refaat et al's study [45] is classified as direct/coupled because COMP is biologically active.

Comparison	Main finding	Interpretation
Higher nominal dose [4,8,26,38,39]	Mass changed together with one or more of product loading concentration, loaded-carrier volume, additional-graft volume or configuration, delivery, anatomy, or practice.	The comparison does not isolate the effect of milligrams; the direction of change in ATU dose concentration depends on the relative changes in mass and final implanted graft bulk volume.
Same mass, different final concentration [40]	The same 3 mg failed at 0.6 mg/mL but achieved bilateral fusion at 1.5 mg/mL.	Concentration changed the biological effect at equal mass; total mass was not sufficient.
Same nominal rhBMP-2 mass, different release, delivery, carrier, retention, or biological presentation [19-23,41-45]	The relevant pairwise comparisons held nominal rhBMP-2 mass constant and produced different fusion, ectopic-bone, or spatial outcomes when release, delivery, carrier, retention, or biological presentation changed.	Delivery and biological presentation can alter outcomes at equal nominal mass; these studies do not identify a single replacement exposure metric.
Lower mass in a smaller cervical ATU [26-31]	Clinically important local events occurred below many lumbar dose ranges.	These comparisons are consistent with anatomical context modifying the implications of a given nominal mass.
Very high mass in open posterolateral fusion [10,26,32]	A consistent monotonic complication gradient was not detected.	Treated area, dispersal, and containment matter.
Low mass in smaller animal ATUs [12,33-39]	Characteristic osteolytic biology occurred far below human patient totals.	Cross-species translation should consider local graft volume, configuration, and anatomy rather than patient total mass alone.

Why does anatomy change the local meaning of mass?

Cervical and Lumbar Interbody Fusion

Anterior cervical use illustrates why lower mass does not necessarily mean lower local exposure. Dysphagia, prevertebral swelling, airway compromise, endplate resorption, subsidence, and heterotopic bone have been reported at per-level masses below many lumbar dose regimens [27-31]. All else equal, and to the extent that the smaller anatomy contains a smaller implanted graft construct, a given mass produces a higher nominal ATU dose concentration. The cervical implant lies adjacent to prevertebral soft tissues and neural structures; consequently, relatively limited swelling or ectopic bone may be clinically consequential [27-31].

This is a transportability argument rather than a pooled cervical-to-lumbar risk comparison. The procedures differ in approach, cage design, containment, drainage, number of levels, ascertainment, and era of practice. Those differences prevent a simple numerical risk ratio, but they do not support a universal threshold expressed only in milligrams. Local comparisons should therefore report mass per level, product loading concentration, total graft volume and composition, placement, containment, and anatomical susceptibility.

Open Posterolateral Fusion

Open posterolateral lumbar fusion provides the complementary example. The exploratory procedure-level meta-analysis did not detect a monotonic dose-complication relationship even at nominal masses of approximately 35 to 40 mg per level [26]. A cohort of 559 patients with posterolateral lumbar fusion spanning 1.5 to 24 mg per level likewise found no dose-related increase in infection, seroma, or bone overgrowth; fusion was less reliable below approximately 6 mg per level, but increasing dose above that range did not improve union [32]. These findings do not define an unlimited safe dose. They indicate that high total mass alone was not associated with a reproducible complication gradient in these datasets.

One plausible explanation is spatial distribution. Unlike an interbody cage, the posterolateral graft bed is not a fixed-volume chamber, and graft can be distributed across a broader decorticated area [10,26,32]. Consequently, greater total mass need not imply a proportionate increase in local concentration or areal loading. Practice data are consistent with location-specific behavior: interbody dose fell markedly over time, whereas posterolateral-gutter amounts changed much less [10]. The evidence does not prove that concentration is the sole determinant, but it is difficult to reconcile with a universal milligram threshold that ignores treated area and containment.

Sheep and Human Interbody Fusion

Large-animal translation raises the same issue. Characteristic peri-implant osteoclastic resorption and osteolytic voids have been produced in sheep at sub-milligram to low-milligram masses [38,39], and transient resorption occurred in a nonhuman primate core-defect model at 0.36 mg delivered at 1.5 mg/mL [33]. A high-dose cervical sheep model also produced vertebral resorption and heterotopic bone [34]. These findings indicate that a large human patient total mass is not required to produce qualitatively similar local biological responses in preclinical models.

Ovine and human spines are not geometrically interchangeable [12,35-37]. Comparative anatomy and methodological reviews document differences in disc-space dimensions, cage fit, endplate coverage, and the relationship between implant and recipient anatomy [12,35-37]. Given these geometric differences, placing a human patient's total mass into one smaller animal graft space may produce a substantially higher mass per graft volume and may not reproduce the clinical local configuration [12,35-37]. Overload can be useful as an intentional stress test, but it should not be described as local exposure matching.

Regulatory case study: the 2026 Medtronic Infuse TLIF program

A recent regulatory example illustrates many of the practical reporting limitations discussed above. The active groups in the 2026 Medtronic Infuse transforaminal lumbar interbody fusion program received 2.1 mg in 1.4 mL or 4.2 mg in 2.8 mL of loaded sponge per treated level; both used the same 1.5 mg/mL product loading concentration [4]. All subjects received local autograft, whereas mineralized cancellous allograft was optional [4]. In one-level subjects, mean local autograft volumes were 6.7 and 6.1 mL in the 2.1- and 4.2-mg groups, respectively, demonstrating that non-rhBMP-2 graft comprised a substantial component of the reported construct [4].

The SSED reports treatment-arm mean graft components but does not provide patient-level graft allocation or divide graft volumes among the cage, the Infuse-containing extracage region, and the remainder of the disc space [4]. The allograft-recipient denominators are also not reported. Patient-level or absolute arm-level ATU dose concentrations therefore cannot be reconstructed reliably from the SSED. The detailed graft-volume and denominator considerations are provided in Appendix B.

Assuming broadly comparable total graft-volume distributions between treatment arms, doubling rhBMP-2 mass would also approximately double the nominal whole-ATU concentration. Some one-level outcomes were numerically higher in the 4.2-mg group, but patient-level graft configuration, compartmental distribution, and actual graft volumes were not reported, event numbers were small, and broader procedural safety outcomes were not consistently ordered between the one- and two-level cohorts [4]. Consequently, the program provides useful scale context and illustrates the limitations of interpreting total mass in isolation; it provides a testable hypothesis but does not validate ATU dose concentration as an independent predictor of adverse events.

Operational reporting of implanted graft volume and configuration

Future interbody studies should report the implanted bulk volume of every graft component in each ATU: rhBMP-2-loaded carrier, local autograft, allograft, ceramic, demineralized bone matrix, and any other graft. Material prepared but not implanted should be recorded separately and excluded from the implanted-volume denominator. For quantitative reporting, the method used to determine implanted bulk volume should be prespecified. For particulate graft, authors should state whether volume was measured loose-filled or after packing; for hydrated or compressible carriers, the reported value should state whether it represents nominal prepared volume or an estimated implanted volume. Where components interpenetrate or their implanted volumes cannot be measured independently, component volumes should not be assumed to be simply additive, and the associated uncertainty should be reported. Because the ACS is soft and pliable and may be compressed during implantation [3,14], nominal kit volume should be distinguished from its estimated implanted bulk-volume contribution. Cage dimensions and internal graft volume, final expansion where applicable, the intended extracage graft bed, and placement relative to endplates, annular defects, and neural structures should be reported where possible. In multilevel surgery, each treated level should be treated as a separate ATU, while patient total mass should also be reported for cumulative-dose assessment.

This information is central to interpreting a construct-average measure. Clinical studies have associated fusion with both implanted graft volume and its spatial distribution [11,13]. Reducing graft fill would alter the treatment construct rather than isolate rhBMP-2 exposure. Total implanted graft volume therefore provides the denominator for whole-ATU nominal concentration, but its biological interpretation depends on how the rhBMP-2-loaded carrier and other graft components are mixed, layered, compressed, or spatially separated.

ATU dose concentration is calculated by dividing the rhBMP-2 mass in one ATU by the total implanted graft bulk volume determined using the prespecified measurement method. Component volumes should be reported separately because nominal kit volume may not represent the carrier's implanted bulk-volume contribution after handling and implantation [3,14]. Where implanted carrier volume cannot be estimated reproducibly, the calculation can be presented with and without nominal carrier volume as a sensitivity analysis. For expandable cages, final device geometry and implanted graft bulk volume should be reported rather than assuming nominal device capacity. Where cage and extracage graft are spatially separate, their locations and component volumes should be reported separately; the whole-ATU value should not be interpreted as a homogeneous local concentration.

Where the rhBMP-2-containing carrier occupies a discrete compartment, product loading concentration, carrier volume, and the spatial relationship to surrounding graft and vulnerable tissues should be reported alongside the whole-ATU construct average and may be more locally informative. ATU dose concentration should be reported per subject and per level and remains a proposed nominal construct-average descriptor rather than a measured tissue concentration [19-26,46-51].

Implications for study design and nonclinical translation

Local and systemic safety are different but related domains. Patient total mass remains an important cumulative-dose descriptor for systemic assessment and should be interpreted with release, biodistribution, and pharmacokinetic data [3,16-18]. Local safety studies should report mass per level, product loading concentration, the proposed ATU dose concentration, graft-component volumes, configuration, placement, containment, and release rather than relying on a single exposure number. General medical-device animal-study and biological-evaluation principles support selecting clinically relevant anatomy and application and assessing both local and systemic endpoints [17,18].

A clinically faithful interbody experiment should keep anatomy, cage geometry, final graft fill, placement, and containment as constant as possible. Equal-mass groups can then vary product loading concentration, carrier configuration, release, or spatial presentation; such comparisons test whether mass alone is sufficient but do not test ATU dose concentration. Testing ATU dose concentration specifically requires rhBMP-2 mass and final implanted graft bulk volume to vary independently, ideally in a factorial design and within clinically plausible graft-fill ranges.

Different outcomes at equal mass and equal final graft fill would show that delivery or configuration modifies biological effect; they would not, by themselves, validate ATU dose concentration. Independent evaluation of the proposed denominator requires patient-level or experimental variation in final implanted graft bulk volume. Prespecified outcomes should distinguish the intensity and extent of the local response, containment failure, off-target bone, osteolysis, inflammation, and systemic exposure.

For translation, the maximum human patient total should not automatically be placed into one smaller animal ATU and described as a clinical local-exposure match. Local exposure comparisons should consider ATU dose concentration together with graft composition, placement, containment, delivery, and species-specific anatomy [12,35-37], while recognizing that no single construct-average metric has been validated as the correct translation rule. As a study-design recommendation, cumulative or systemic questions could be addressed by distributing exposure across multiple animal units where feasible or by dedicated pharmacokinetic and toxicology arms. A single-unit overload can still be used as an intentional stress test, provided it is clearly identified as such.

ATU dose concentration is not a tissue concentration

The ATU framework is consistent with known rhBMP-2 biology but should not be equated with measured tissue exposure. Clinical and preclinical studies associate exposure with osteolysis or resorption, heterotopic bone, local cytokine responses, and neural inflammation, although clinical radiculitis has not shown a consistent dose relationship [46-51]. rhBMP-2 released from a collagen sponge can remain osteoinductive [24], and controlled-delivery strategies can change retention, release, and the spatial distribution of bone while preserving or improving bone formation [16,19-23,25]. ATU dose concentration is therefore a proposed starting descriptor of the completed construct; realized tissue exposure is further shaped by release kinetics, spatial distribution, placement, containment, and anatomy.

Total mass remains important because it defines the nominal molecular reservoir [3]. How that reservoir translates into the duration, spatial extent, and systemic distribution of exposure depends on carrier retention, delivery, and biodistribution [16-25]. A higher whole-ATU nominal concentration could plausibly alter local concentration gradients, but retention, release, spatial configuration, placement, containment, and tissue transport determine how far and how long protein reaches surrounding tissue [3,16,19-25]. The purpose of the ATU framework is therefore not to replace mass with a single alternative number, but to describe the treatment construct more completely. The low-dose literature is consistent with the clinical principle of using the minimum effective treatment package [52].

Limitations

This is a structured narrative review rather than a systematic review. The contemporary PubMed search returned 253 records: 24 materially informed the narrative synthesis, 25 were relevant to the exposure framework but were not individually discussed, 85 were eligible but redundant, and 119 were excluded at title/abstract screening. The conclusion that total mass was not isolated as a sufficient general descriptor of local exposure is also informed by targeted foundational, mechanistic, anatomical, graft-volume, regulatory, and post-search sources. Selection of these additional sources was purpose-driven rather than systematic, so literature completeness cannot be assured. Appendix A summarizes the source type or study design, evidentiary role, proximity to human spinal application, and principal limitations or internal-validity considerations for each principal source or study that materially informed the exposure synthesis. Because screening, classification, source selection, extraction, appraisal, and synthesis were performed by one author and were not independently duplicated, selection and interpretation bias remain possible.

The evidence base is heterogeneous. Barnes provides direct preclinical spinal evidence that biological efficacy can differ at the same mass. Similarly, other studies include matched-nominal-mass carrier or release comparisons, some within studies that also included separate dose-response arms, indicating that delivery can alter ectopic or heterotopic bone distribution. Those carrier studies are nonspinal bone-defect models and do not estimate clinical spinal adverse-event rates [19-23,40]. Cross-procedure, cross-anatomical, and cross-species comparisons differ in approach, containment, tissue susceptibility, endpoint, and ascertainment. They are therefore contextual and hypothesis-generating: they test the transportability of a mass-only description but cannot establish that ATU dose concentration explains the observed differences or define a universal safe value.

No clinical dataset in the reviewed evidence reported the patient-level mass, graft-component volumes, spatial configuration, placement, and adverse outcomes needed to test whether ATU dose concentration independently predicts events or adds information beyond total mass and product loading concentration. The transforaminal lumbar interbody fusion SSED reports only treatment-arm mean graft components, does not state the allograft-recipient denominators, and does not allocate graft between cage and extracage compartments [4]. Absolute patient-level ATU dose concentration therefore cannot be reconstructed. Grafting-material-related serious adverse event counts were small, and the broader procedure-related endpoint did not show a consistent ordering between one- and two-level cohorts [4]. ATU dose concentration should therefore be treated as a proposed exposure descriptor and testable hypothesis, not a validated clinical risk score.

ATU dose concentration is a nominal average across the implanted graft construct, not a measured tissue concentration. Total graft volume is an anatomically interpretable denominator for whole-construct normalization, but the reviewed evidence does not establish that every implanted graft component contributes equally to biological dilution. The denominator is implanted graft volume rather than anatomical tissue volume, surface area, distance to susceptible structures, or accessible fluid space; the term ATU therefore denotes the level-specific treatment construct rather than a measured anatomical concentration. Actual fill, packing, compression, unused material, cage expansion, extracage placement, mixing, and spatial separation may vary and should be measured using prespecified methods or reported as uncertain rather than assumed. The available graft-bearing volume within an ATU also varies between patients [12]. Within a standardized product, procedure, anatomy, and placement, nominal mass may remain a useful empirical predictor. The present framework is intended to make these additional exposure variables explicit so that their incremental predictive value can be tested prospectively.

## Conclusions

Across the clinical, preclinical, and regulatory evidence reviewed, total rhBMP-2 mass alone was not shown to be a sufficient general descriptor of local exposure when graft configuration, delivery, or anatomy differed. Higher nominal-dose comparisons usually altered other exposure variables, whereas equal-mass experiments produced different biological or spatial outcomes when concentration or delivery changed. These findings support reporting the completed graft construct and local anatomical context alongside mass, but they do not identify a single alternative metric that fully captures realized tissue exposure.

Product loading concentration describes rhBMP-2 on its carrier before implantation. ATU dose concentration is a proposed whole-construct nominal measure that relates mass at one treated level to the total graft volume implanted at that level. It may provide useful complementary information by making graft volume and treatment geometry explicit, but the reviewed evidence does not establish total graft volume as the biologically correct denominator or validate ATU dose concentration as an independent predictor of clinical complications. Future studies should report mass per level, product loading concentration, graft-component volumes, configuration, placement, containment, release, and the proposed ATU dose concentration with patient-level outcomes so that its incremental predictive value can be tested. Patient total mass should continue to be reported for cumulative-dose and systemic assessment.

## Appendices

Appendix A: sources materially informing the narrative synthesis 

**Table 3:** Study-level evidence table for principal sources materially informing the exposure synthesis Evidence category definitions: direct evidence holds mass constant while another exposure variable changes; coupled evidence changes mass together with one or more other exposure variables; contextual evidence informs transportability or mechanism but cannot validate ATU dose concentration as an independent clinical predictor. "Clinical directness" denotes proximity to human spinal application and is not a formal risk-of-bias, quality, or certainty grade. Source-identification routes: references 3-7, 11-23, 25-26, 31, 33, 35-37, and 39-40 were identified outside the contemporary PubMed export through backward citation searching and targeted retrieval of regulatory, foundational, mechanistic, anatomical, graft-volume, and delivery evidence. The complete record-level disposition is available upon reasonable request to the corresponding author (jvonbenecke@locatebio.com). TLIF: transforaminal lumbar interbody fusion; PLIF: posterior lumbar interbody fusion; SSED: Summary of Safety and Effectiveness Data; ATU: anatomic treatment unit; rhBMP-2: recombinant human bone morphogenetic protein-2; ACDF: anterior cervical discectomy and fusion; FDA: Food and Drug Administration

Ref.	Source type/design	Anatomy/model	Evidence category	Exposure variables relevant to review	Clinical directness	Principal limitation/internal-validity consideration
3	FDA regulatory SSED	Human ALIF/product	Contextual	Loading concentration, carrier, residence time	High	Regulatory summary; not designed to compare ATU metrics
4	FDA regulatory SSED	Human TLIF	Coupled	Mass, loaded-sponge volume, autograft/allograft, placement	High	No patient-level graft allocation; allograft denominator and compartment volumes unavailable
8	Longitudinal clinical cohort	Mixed spinal arthrodesis	Coupled	Dose per level; practice changes over time	High	Graft volumes/configuration not reported; temporal confounding
10	Institutional clinical practice cohort	Posterior fusion	Contextual	Dose trends by location	Moderate	Practice-pattern data; not exposure-response validation
11	Clinical cohort	MIS TLIF	Contextual	Graft mixture ratio and volume	Moderate	Does not test rhBMP-2 safety metric directly
12	Anatomical study	Human intervertebral space	Contextual	Intervertebral volume/geometry	Moderate	Anatomical measurements, not rhBMP-2 outcomes
13	Clinical imaging study	TLIF	Contextual	Spatial graft distribution	Moderate	Fusion distribution rather than rhBMP-2 exposure
14	Carrier characterization	In vitro/biomaterial	Contextual	ACS handling and compression	Moderate	Does not measure in vivo clinical exposure
16	Preclinical pharmacokinetics	Rat ectopic model	Contextual	Carrier retention and rhBMP-2 pharmacokinetics	Low-moderate	Nonspinal small-animal model
19	Preclinical carrier/delivery study	Nonspinal mandible defect	Direct	Same nominal rhBMP-2 mass across carrier comparisons; kerateine (reduced keratin) delivery versus collagen-sponge control	Low-moderate	Nonspinal small-animal model; carrier changes multiple material properties; ectopic/regeneration endpoints do not estimate spinal clinical safety
20	Preclinical carrier/delivery study	Nonspinal critical bone defect	Direct	Same high-dose BMP-2 delivered by different vehicles	Low-moderate	Nonspinal defect model; high-dose context and delivery vehicle changes multiple properties
21	Preclinical controlled-release/ retention study	Nonspinal bone defect	Direct	Same nominal BMP-2 dose with heparin-mediated retention/release modification	Low-moderate	Nonspinal model; biomaterial-retention mechanism does not validate a clinical spinal safety metric
22	Preclinical delivery-strategy study	Complex extremity trauma/bone defect	Direct	Same BMP-2 dose with delivery strategy altered; local bone and systemic immune outcomes assessed	Low-moderate	Nonspinal trauma model; local and systemic endpoints do not estimate spinal clinical complication rates
23	Preclinical dose/delivery study	Nonspinal segmental bone-defect model	Direct/coupled	Matched-dose carrier comparisons plus separate dose-response arms	Low-moderate	Nonspinal model; includes dose-response arms and multiple delivery variables; not clinical spinal safety
24	Preclinical release study (post-search source)	In vivo proxy	Contextual	Released rhBMP-2 osteoinductivity	Low-moderate	Proxy for burst release; not clinical safety
26	Exploratory meta-analysis	Multiple spinal procedures	Coupled/contextual	Dose, procedure, placement	High	Study-level heterogeneity; exposure variables incompletely reported
27	Systematic review/meta-analysis	Anterior cervical fusion	Contextual	Cervical mass, complications, and procedure-specific safety context	High	Cross-study procedural and ascertainment heterogeneity; does not test ATU denominator
28	Clinical cohort/case series	Anterior cervical fusion	Contextual	Low-dose rhBMP-2 cervical use; dose and local safety context	High	Clinical cervical source but construct-level graft volumes and denominator not reported
29	Prospective clinical comparison	ACDF with and without rhBMP-2	Contextual	rhBMP-2 exposure and dysphagia/local soft-tissue outcome context	High	Not designed to test an exposure metric; dose, containment, and graft-volume data incomplete
30	Clinical cohort	Anterior cervical fusion	Contextual	Contained-delivery route, postoperative steroids, and low-dose rhBMP-2	High	Coupled intervention and practice factors; no construct-level ATU denominator
31	Clinical adverse-event series	Anterior cervical fusion	Contextual	High-dose cervical rhBMP-2 and local complications	High	Historical high-dose practice; uncontrolled ascertainment and incomplete construct-level exposure data
32	Clinical cohort	Posterolateral lumbar fusion	Contextual	Dose per level and complications	High	Open graft bed; graft area/volume not standardized
33	Preclinical primate study	Core-defect model	Contextual	0.36 mg at 1.5 mg/mL; resorption	Moderate	Nonspinal defect and species differences
34	Preclinical ovine study	Cervical fusion	Contextual	High-dose rhBMP-2; resorption/heterotopic bone	Moderate	Different anatomy and exposure configuration
35	Methodological/anatomical review	Ovine lumbar interbody fusion	Contextual	Ovine interbody geometry, cage fit, graft-space relationships	Moderate	Methodological review; no direct rhBMP-2 exposure-response test
36	Comparative anatomy study	Sheep and human spine	Contextual	Spine geometry and disc/endplate relationships	Moderate	Anatomical comparison only; no rhBMP-2 outcomes
37	Systematic comparative anatomy review	Large-animal and human spine	Contextual	Species geometry and spine-model selection	Moderate	Anatomical/methodological review; no direct rhBMP-2 exposure-response test
38	Preclinical ovine interbody study	Lumbar interbody fusion	Coupled	Mass and loading concentration changed together	Moderate-high	Cannot isolate mass from concentration
39	Preclinical ovine interbody study	Anterior lumbar interbody fusion	Coupled	Mass and loading concentration changed together	Moderate-high	Cannot isolate mass from concentration
40	Preclinical nonhuman primate study	Posterolateral fusion	Direct	Same mass; different final concentration/carrier configuration	Moderate	Efficacy endpoint rather than adverse-event endpoint
41	Preclinical controlled-release study	Rabbit interbody fusion chamber	Direct	Same 100 µg rhBMP-2; controlled-release polyelectrolyte complex versus ACS	Moderate	Small-animal model; fusion and molecular endpoints rather than clinical safety
42	Preclinical split-side delivery study	Posterolateral spinal fusion animal model	Direct	Same 0.53 mg per side with same ACS/loading preparation; open versus percutaneous placement	Moderate	Delivery route changes access and spatial placement; preclinical efficacy and ectopic-bone endpoints
43	Preclinical carrier comparison	Rat posterolateral fusion	Direct	Matched 0.2, 2.0, and 20 µg per side on collagen versus BioMim-PDA	Low-moderate	Small-animal model; scaffold changes multiple material properties and was not uniformly superior at every endpoint
44	Preclinical retention/release study	Rat posterolateral fusion	Direct	Same 5 µg free rhBMP-2 versus calcium-binding PLGA/PBMP microparticles	Low-moderate	Small groups; free-protein versus particle delivery; preclinical endpoint
45	Preclinical BMP-binding cofactor study	Rat spinal fusion	Direct/coupled	Same 2 µg rhBMP-2 with or without COMP; 10 µg comparator	Low-moderate	COMP is biologically active, so the comparison does not isolate a simple carrier or concentration effect
46	Clinical CT review	TLIF	Contextual	Heterotopic ossification after rhBMP-2; dose/placement incompletely captured	High	Retrospective CT review; construct-level mass, graft-volume, and spatial-exposure data incomplete
47	Clinical retrospective review	PLIF	Contextual	rhBMP-2 use, complications, radiculitis/heterotopic bone context	High	Retrospective design; exposure data and construct-level denominator incomplete
48	Clinical cohort/retrospective analysis	TLIF	Contextual	rhBMP-2-induced radiculitis and relationship to dose	High	Clinical dose-radiculitis analysis, but graft configuration and ATU denominator unavailable
49	Mechanistic/preclinical study	Cell/spinal model	Contextual	Osteoclast-mediated osteolysis mechanisms	Low-moderate	Mechanistic endpoints do not validate a clinical exposure metric
50	Mechanistic clinical study	Spinal fusion/local tissue response	Contextual	Local cytokine and growth-factor response to rhBMP-2	Low-moderate	Mechanistic/local inflammatory endpoints do not validate a clinical exposure metric
51	Preclinical rodent nerve model	Rodent spinal nerve model	Contextual	Dose-dependent neural inflammatory response	Low-moderate	Rodent model and mechanistic neural inflammation endpoint; not clinical risk validation
52	Narrative review	Spinal fusion	Contextual	Low-dose clinical literature	moderate	Secondary source; not independent validation

Appendix B: 2026 Infuse TLIF SSED graft-volume and denominator considerations

The SSED reports Infuse placement outside the interbody device in the anterior and contralateral disc space, surrounded by autograft and optionally supplemented with allograft; all grafting was confined to the interbody space [4]. Reported graft volumes were not allocated among the cage, the Infuse-containing extracage region, and the remainder of the disc space. Mean allograft volumes were reported without the number of contributing recipients, and the pooled allograft means are not the sample-size-weighted averages of the regional means. The rounded regional and pooled values constrain, but do not uniquely determine, recipient counts; autograft and allograft means therefore should not be added to estimate an arm-wide denominator. These reporting limitations preclude reliable reconstruction of patient-level or absolute arm-level ATU dose concentration and of the nominal concentration within the smaller Infuse-containing compartment.
